# Genome sequence of the Listia angolensis microsymbiont *Microvirga lotononidis* strain WSM3557^T^

**DOI:** 10.4056/sigs.4548266

**Published:** 2013-12-31

**Authors:** Wayne Reeve, Julie Ardley, Rui Tian, Sofie De Meyer, Jason Terpolilli, Vanessa Melino, Ravi Tiwari, Ronald Yates, Graham O’Hara, John Howieson, Mohamed Ninawi, Xiaojing Zhang, David Bruce, Chris Detter, Roxanne Tapia, Cliff Han, Chia-Lin Wei, Marcel Huntemann, James Han, I-Min Chen, Konstantinos Mavromatis, Victor Markowitz, Ernest Szeto, Natalia Ivanova, Ioanna Pagani, Amrita Pati, Lynne Goodwin, Tanja Woyke, Nikos Kyrpides

**Affiliations:** 1 Centre for Rhizobium Studies, Murdoch University, Western Australia, Australia; 2 Department of Agriculture and Food, Western Australia, Australia; 3 Los Alamos National Laboratory, Bioscience Division, Los Alamos, New Mexico, USA; 4 DOE Joint Genome Institute, Walnut Creek, California, USA; 5 Biological Data Management and Technology Center, Lawrence Berkeley National Laboratory, Berkeley, California, USA

**Keywords:** root-nodule bacteria, nitrogen fixation, symbiotic specificity, *Alphaproteobacteria*

## Abstract

*Microvirga lotononidis* is a recently described species of root-nodule bacteria that is an effective nitrogen- (N_2_) fixing microsymbiont of the symbiotically specific African legume *Listia angolensis* (Welw. ex Bak.) B.-E. van Wyk & Boatwr. *M. lotononidis* possesses several properties that are unusual in root-nodule bacteria, including pigmentation and the ability to grow at temperatures of up to 45°C. Strain WSM3557^T^ is an aerobic, motile, Gram-negative, non-spore-forming rod isolated from a *L. angolensis* root nodule collected in Chipata, Zambia in 1963. This is the first report of a complete genome sequence for the genus *Microvirga*. Here we describe the features of *Microvirga lotononidis* strain WSM3557^T^, together with genome sequence information and annotation. The 7,082,538 high-quality-draft genome is arranged in 18 scaffolds of 104 contigs, contains 6,956 protein-coding genes and 84 RNA-only encoding genes, and is one of 20 rhizobial genomes sequenced as part of the DOE Joint Genome Institute 2010 Community Sequencing Program.

## Introduction

Legume-rhizobia symbioses are important components of southern Australian agricultural systems, in which symbiotic N_2_-fixation provides a significant amount of the nitrogen input that is required to boost food and animal production [1,2]. Traditionally, pasture legumes have been Mediterranean annuals such as medics and subterranean clover [3]. However, recent changes to the rainfall patterns in south-western Western Australia, resulting in a 10-20% decrease in annual rainfall [4], have adversely affected production from these annual legumes. Researchers are therefore seeking to introduce alternative perennial legume species and associated rhizobia that are better adapted to the arid climate and acid, infertile soils found in these systems [2]. Among the perennial, herbaceous forage legumes selected for further study are several species within the papilionoid legume clade *Lotononis sensu lato.*

*Lotononis s. l.* is grouped within tribe *Crotalarieae*, has a centre of origin in South Africa and consists of some 150 species, divided into 15 sections [5]. The taxonomy has recently been revised and the three distinct clades within *Lotononis s. l*. are now recognized at the generic level as *Listia*, *Leobordea* and *Lotononis s. s.* [6]. Species within the genus *Listia* are of agronomic interest, as they have potential as perennial pasture legumes that are able to reduce groundwater recharge and assist in preventing dry land salinity in southern Australian agricultural systems [7]. *Listia* spp. produce stoloniferous roots on their lower branches (a characteristic thought to be associated with the seasonally wet habitats where these species are found) [5] and form lupinoid, rather than indeterminate nodules, in response to infection by rhizobia [7,8]. The symbioses between *Listia* species and their associated root-nodule bacteria are highly specific. All studied host species are nodulated by strains of pigmented methylobacteria [7,9,10], except for *Listia angolensis*, which is effectively nodulated only by newly described species of *Microvirga* [11]. *Microvirga lotononidis* strain WSM3557^T^ is the type strain for this species. Here we present a set of preliminary classification and general features for *M. lotononidis* strain WSM3557^T^ together with the description of the genome sequence and annotation.

## Classification and general features

**Figure 1:**
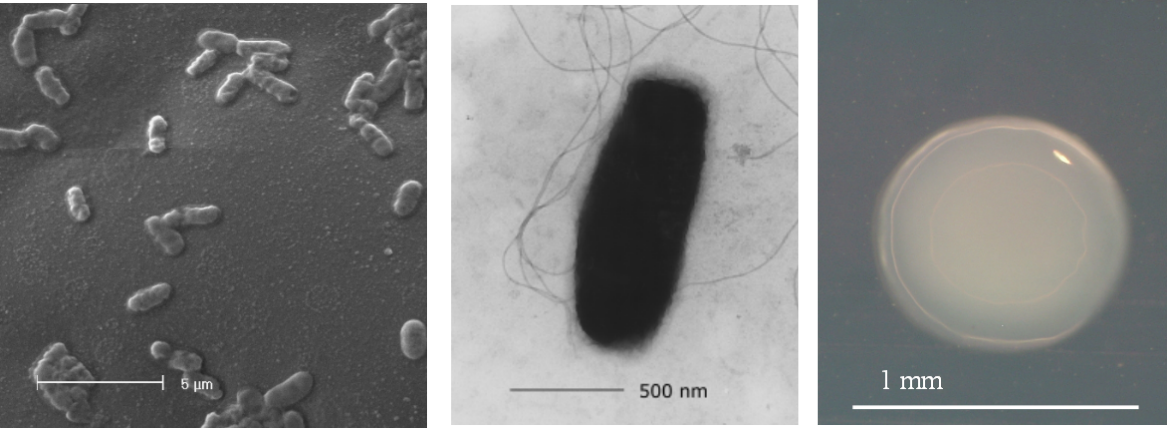
Images of *Microvirga lotononidis* strain WSM3557^T^ using scanning (Left) and transmission (Center) electron microscopy as well as light microscopy to visualize colony morphology on a solid medium (Right).

**Figure 2:**
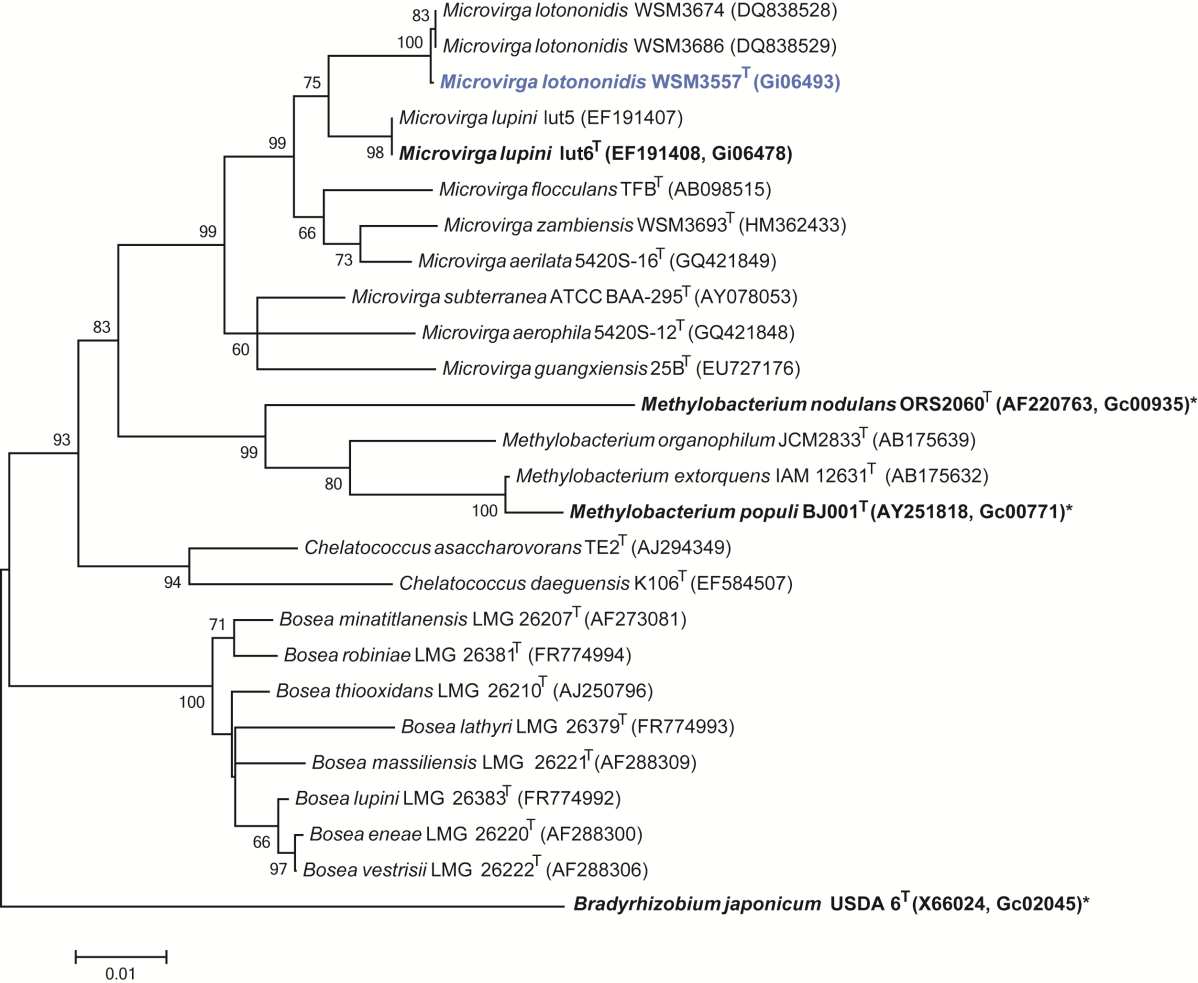
Phylogenetic tree showing the relationships of *Microvirga lotononidis* WSM3557^T^ (shown in blue print) with some of the root nodule bacteria in the order *Rhizobiales* based on aligned sequences of the 16S rRNA gene (1,255 bp internal region). All sites were informative and there were no gap-containing sites. Phylogenetic analyses were performed using MEGA, version 5.05 [12]. The tree was built using the maximum likelihood method with the General Time Reversible model. Bootstrap analysis [13] with 500 replicates was performed to assess the support of the clusters. Type strains are indicated with a superscript T. Strains with a genome sequencing project registered in GOLD [14] are in bold print and the GOLD ID is mentioned after the accession number. Published genomes are designated with an asterisk.

*M. lotononidis* strain WSM3557^T^ is a motile, Gram-negative, non-spore-forming rod with one to several flagella (Figure 1, left and center panel). It is a member of the family *Methylobacteriaceae* in the class *Alphaproteobacteria* (Figure 2). WSM3557^T^ is fast growing, forming 0.5-1.5 mm diameter colonies within 2-3 days. It is moderately thermophilic and has a mean generation time of 1.6 h when grown in broth at the optimum growth temperature of 41°C [15]. WSM3557^T^ is pigmented, an unusual property for rhizobia. Colonies on half Lupin Agar (½LA) [7] are pale pink, opaque, slightly domed, moderately mucoid with smooth margins (Figure 1, right panel). The color develops after several days. WSM3557^T^ is able to tolerate a pH range between 6.0 and 9.5 [11]. Carbon source utilization, cellular fatty acid profiles, polar lipid analysis and respiratory lipoquinone analysis have been described previously [11]. Minimum Information about the Genome Sequence (MIGS) is provided in Table 1.

**Table 1:** Classification and general features of *Microvirga lotononidis*. strain WSM3557^T^ in according to the MIGS recommendations [16,17].

**MIGS ID**	**Property**	**Term**	**Evidence code**
	Current classification	Domain *Bacteria*	TAS [17]
		Phylum *Proteobacteria*	TAS [18]
		Class *Alphaproteobacteria*	TAS [18]
		Order *Rhizobiales*	TAS [19]
		Family *Methylobacteriaceae*	TAS [20]
		Genus *Microvirga*	TAS [21]
		Species *Microvirga lotononidis*	TAS [15]
	
	Gram stain	Negative	TAS [15]
	Cell shape	Rod	TAS [15]
	Motility	Motile	TAS [15]
	Sporulation	Non-sporulating	TAS [15]
	Temperature range	Mesophile	TAS [15]
	Optimum temperature	41°C	TAS [15]
	Salinity	Non-halophile	TAS [15]
MIGS-22	Oxygen requirement	Aerobic	TAS [15]
	Carbon source	L-arabinose, D-cellobiose, D-fructose, D-glucose, glycerol, D-mannitol, acetate, succinate & glutamate	TAS [15]
	Energy source	Chemoorganotroph	TAS [15]
MIGS-6	Habitat	Soil, root nodule on host	TAS [15]
MIGS-15	Biotic relationship	Free living, symbiotic	TAS [15]
MIGS-14	Pathogenicity	Non-pathogenic	NAS
	Biosafety level	1	NAS
	Isolation	Root nodule of *Listia angolensis*	TAS [15]
MIGS-4	Geographic location	Chipata, Zambia	TAS [15]
MIGS-5	Nodule collection date	April 1963	TAS [15]
MIGS-4.1	Longitude	32.63	TAS [15]
MIGS-4.2	Latitude	-13 65	TAS [15]
MIGS-4.3	Depth	Not recorded	
MIGS-4.4	Altitude	1000	IDA

Evidence codes – IDA: Inferred from Direct Assay; TAS: Traceable Author Statement (i.e., a direct report exists in the literature); NAS: Non-traceable Author Statement (i.e., not directly observed for the living, isolated sample, but based on a generally accepted property for the species, or anecdotal evidence). These evidence codes are from the Gene Ontology project [22].

### Symbiotaxonomy

*M. lotononidis* strain WSM3557^T^ nodulates (Nod^+^) and fixes N_2_ effectively (Fix^+^) with *Listia angolensis*; nodulates and is partially effective on *Leobordea platycarpa, Leobordea bolusii* and *Lotononis crumanina* and nodulates but is unable to fix N_2_ (Nod+, Fix-) with *Leobordea longiflora, Leobordea stipulosa* and *Lotononis falcata* [8]. It forms occasional ineffective nodules with *Phaseolus vulgaris*, but is unable to nodulate *Crotalaria juncea, Indigofera patens, Lotus corniculatus, Lupinus angustifolius,* or *Macroptilium atropurpureum* [11].

## Genome sequencing and annotation

### Genome project history

This organism was selected for sequencing on the basis of its environmental and agricultural relevance to issues in global carbon cycling, alternative energy production, and biogeochemical importance, and is part of the Community Sequencing Program at the U.S. Department of Energy, Joint Genome Institute (JGI) for projects of relevance to agency missions. The genome project is deposited in the Genomes OnLine Database [14] and an improved-high-quality-draft genome sequence in IMG. Sequencing, finishing and annotation were performed by the JGI. A summary of the project information is shown in Table 2.

**Table 2:** Genome sequencing project information for *M. lotononidis* WSM3557^T^

**MIGS ID**	**Property**	**Term**
MIGS-31	Finishing quality	Improved high quality draft
MIGS-28	Libraries used	Illumina GAii shotgun and paired end 454 libraries
MIGS-29	Sequencing platforms	Illumina GAii and 454 GS FLX Titanium technologies
MIGS-31.2	Sequencing coverage	8.3× 454 paired end, 300× Illumina
MIGS-30	Assemblers	Velvet, version 1.0.13; Newbler, version 2.3- PreRelease-6/30/2009; phrap, version SPS - 4.24
MIGS-32	Gene calling method	Prodigal
	GOLD ID	Gi06493
	NCBI project ID	65303
	Database: IMG	2508501114
	Project relevance	Symbiotic N_2_ fixation, agriculture

### Growth conditions and DNA isolation

*Microvirga lotononidis* WSM3557^T^ was grown to mid-logarithmic phase in TY rich medium [23] on a gyratory shaker at 28°C. DNA was isolated from 60 mL of cells using a CTAB (Cetyl trimethyl ammonium bromide) bacterial genomic DNA isolation method [24].

### Genome sequencing and assembly

The improved high quality draft genome of *Microvirga lotononidis* WSM3557^T^ was generated at the DOE Joint Genome Institute (JGI) using a combination of Illumina [25] and 454 technologies [26]. An Illumina GAii shotgun library comprising 71,475,016 reads totaling 5,432.1 Mb reads and 1 paired end 454 library with an average insert size of 10 Kb which produced 582,107 reads totaling 113.9 Mb of 454 data were generated for this genome. All general aspects of library construction and sequencing performed at the JGI can be found at [24]. The initial draft assembly contained 444 contigs in 1 scaffold. The 454 paired end data was assembled together with Newbler, version 2.3 PreRelease-6/30/2009. The Newbler consensus sequences were computationally shredded into 2 Kb overlapping fake reads (shreds). Illumina sequencing data was assembled with Velvet, version 1.0.13 [27], and the consensus sequences were computationally shredded into 1.5 Kb overlapping fake reads (shreds). The 454 Newbler consensus shreds, the Illumina Velvet consensus shreds and the read pairs in the 454 paired end library using parallel phrap, version SPS - 4.24 (High Performance Software, LLC) were integrated. The software Consed [28-30] was used in the following finishing process. Illumina data was used to correct potential base errors and increase consensus quality, using the software Polisher developed at JGI (Alla Lapidus, unpublished). Possible mis-assemblies were corrected using gapResolution (Cliff Han, unpublished), Dupfinisher [31], or sequencing cloned bridging PCR fragments with subcloning. Gaps between contigs were closed by editing in Consed, by PCR and by Bubble PCR (J-F Cheng, unpublished) primer walks. A total of 303 additional reactions were necessary to close gaps and to raise the quality of the finished sequence. The estimated genome size is 7.2 Mb and the final assembly is based on 59.7 Mb of 454 draft data which provides an average 8.3× coverage of the genome and 2,160 Mb of Illumina draft data which provides an average 300× coverage of the genome.

### Genome annotation

Genes were identified using Prodigal [32] as part of the DOE-JGI Annotation pipeline [33], followed by a round of manual curation using the JGI GenePRIMP pipeline [34]. The predicted CDSs were translated and used to search the National Center for Biotechnology Information (NCBI) non-redundant database, UniProt, TIGRFam, Pfam, PRIAM, KEGG, COG, and InterPro databases. These data sources were combined to assert a product description for each predicted protein. Non-coding genes and miscellaneous features were predicted using tRNAscan-SE [35], RNAMMer [36], Rfam [37], TMHMM [38], and SignalP [39]. Additional gene prediction analyses and functional annotation were performed within the Integrated Microbial Genomes (IMG-ER) platform [40].

## Genome properties

The genome is 7,082,538 nucleotides with 63.00% GC content (Table 3) and comprised of 18 scaffolds (Figures 3a,3b and Figure 3c) of 104 contigs. From a total of 7,040 genes, 6,956 were protein encoding and 84 RNA only encoding genes. The majority of genes (67.64%) were assigned a putative function while the remaining genes were annotated as hypothetical. The distribution of genes into COGs functional categories is presented in Table 4.

**Table 3:** Genome Statistics for *Microvirga lotononidis* WSM3557^T^

**Attribute**	**Value**	**% of Total**
Genome size (bp)	7,082,538	100.00
DNA coding region (bp)	5,991,598	84.60
DNA G+C content (bp)	4,462,203	63.00
Number of scaffolds	18	
Number of contigs	104	
Total genes	7,040	100.00
RNA genes	84	1.19
rRNA operons*	1	
Protein-coding genes	6,956	98.81
Genes with function prediction	4,762	67.64
Genes assigned to COGs	5,117	72.68
Genes assigned Pfam domains	5,358	76.11
Genes with signal peptides	656	9.32
Genes with transmembrane helices	1,480	21.02
CRISPR repeats	0	

*1 full-length and 1 partial 23s rRNA gene, 3 partial 5s rRNA genes

**Figure 3a:**
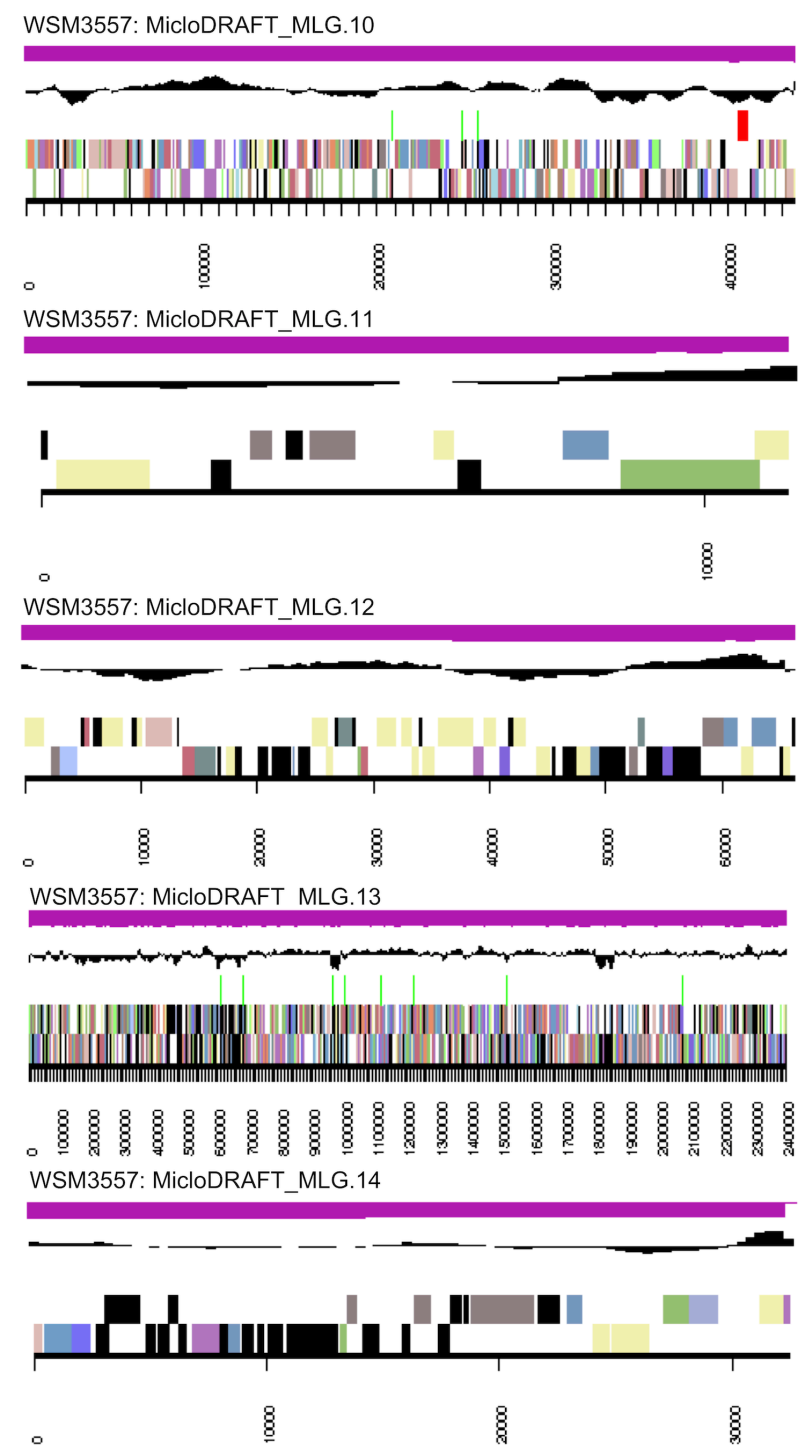
Graphical map of the genome of *M. lotononidis* WSM3557^T^ (scaffolds MLG.1-MLG.9). From the bottom to the top of each scaffold: Genes on forward strand (color by COG categories as denoted by the IMG platform), Genes on reverse strand (color by COG categories), RNA genes (tRNAs green, sRNAs red, other RNAs black), GC content, GC skew.

**Figure 3b:**
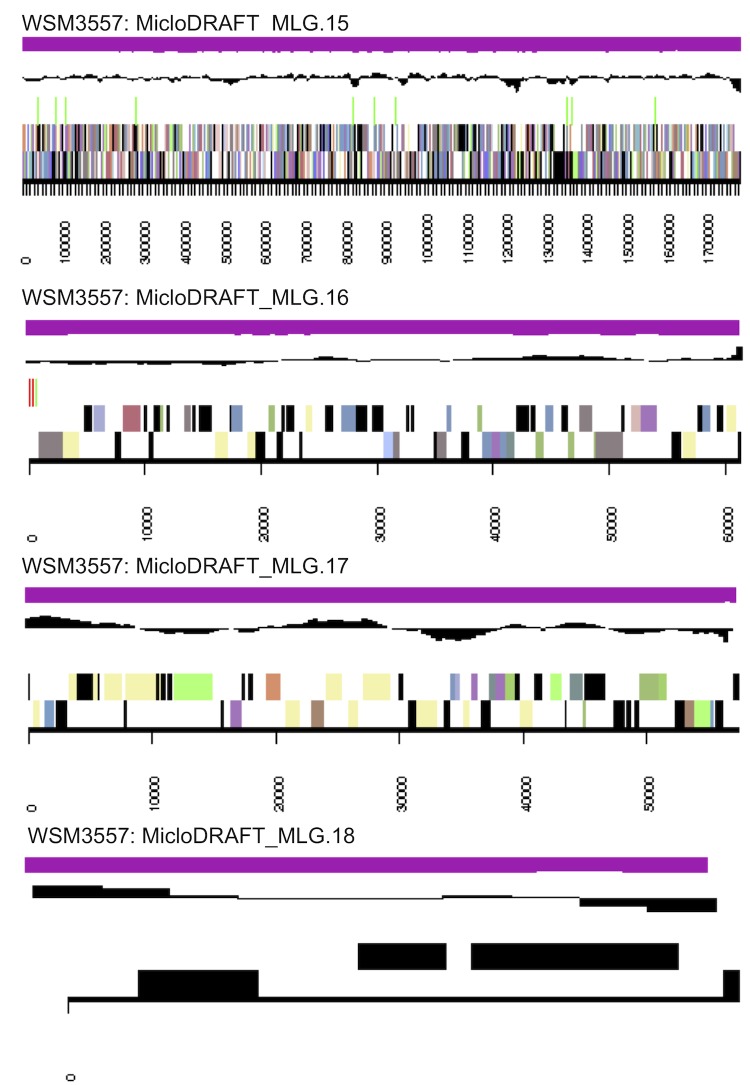
Graphical map of the genome of *M. lotononidis* WSM3557^T^ (scaffolds MLG.10-MLG.18). From the bottom to the top of each scaffold: Genes on forward strand (color by COG categories as denoted by the IMG platform), Genes on reverse strand (color by COG categories), RNA genes (tRNAs green, sRNAs red, other RNAs black), GC content, GC skew.

**Figure 3c:**
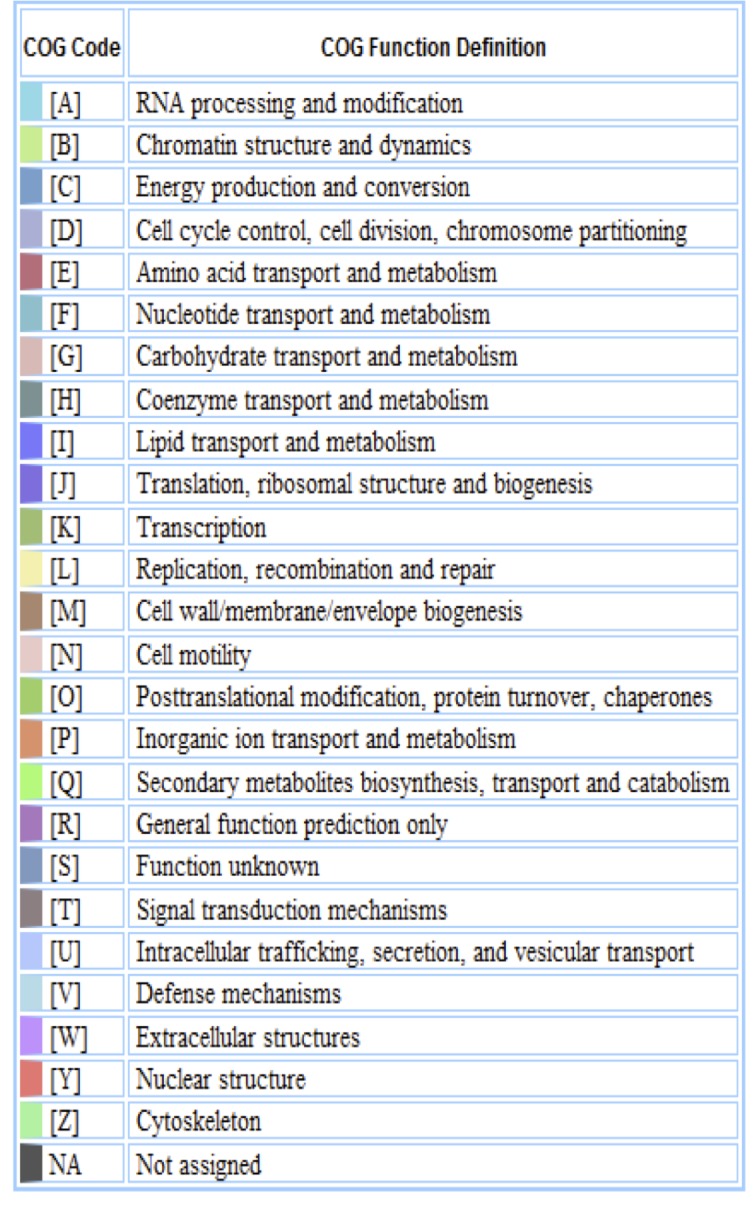
Color code for Figure 3a and 3b.

**Table 4:** Number of protein coding genes of *Microvirga sp.* WSM3557^T^ associated with the general COG functional categories.

**Code**	**Value**	**%age**	**Description**
J	200	3.52	Translation, ribosomal structure and biogenesis
A	1	0.02	RNA processing and modification
K	397	6.98	Transcription
L	431	7.58	Replication, recombination and repair
B	7	0.12	Chromatin structure and dynamics
D	38	0.67	Cell cycle control, mitosis and meiosis
Y	0	0.00	Nuclear structure
V	72	1.27	Defense mechanisms
T	374	6.58	Signal transduction mechanisms
M	254	4.47	Cell wall/membrane biogenesis
N	76	1.34	Cell motility
Z	0	0.00	Cytoskeleton
W	1	0.02	Extracellular structures
U	73	1.28	Intracellular trafficking and secretion
O	178	3.13	Posttranslational modification, protein turnover, chaperones
C	307	5.40	Energy production conversion
G	435	7.65	Carbohydrate transport and metabolism
E	612	10.76	Amino acid transport metabolism
F	105	1.85	Nucleotide transport and metabolism
H	193	3.39	Coenzyme transport and metabolism
I	179	3.15	Lipid transport and metabolism
P	319	5.61	Inorganic ion transport and metabolism
Q	171	3.01	Secondary metabolite biosynthesis, transport and catabolism
R	677	11.91	General function prediction only
S	586	10.31	Function unknown
-	1,923	27.32	Not in COGS
